# Identifying mitochondrial genes and potential biological functions in pre-eclampsia: bioinformatics and experimental insights

**DOI:** 10.3389/fgene.2026.1723592

**Published:** 2026-03-24

**Authors:** Yaohui Wang, Zhixin Du, Liping Yang, Junlin Hou, Xiaolin Li, Mengyang Fan, Chenyang Yu, Jianhua Sun, Li Zhou, Lingling Li, Pengbei Fan

**Affiliations:** 1 School of Traditional Chinese Medicine, Henan University of Chinese Medicine, Zhengzhou, China; 2 Department of Obstetrics and Gynecology, The First Affiliated Hospital of Henan University of Traditional Chinese Medicine, Henan University of Chinese Medicine, Zhengzhou, China; 3 School of Traditional Chinese Medicine, Southern Medical University, Guangzhou, China

**Keywords:** immunometabolism, mitochondria, molecular validation, pre-eclampsia, single cell

## Abstract

**Background:**

Pre-eclampsia (PE) is a specific type of gestational hypertension associated with high morbidity and mortality. This study aims to identify mitochondria-related regulatory molecules in PE through bioinformatics analysis, which will help pinpoint potential therapeutic targets and elucidate potential mechanisms of action in PE.

**Methods:**

This study integrated three PE placental transcriptome datasets (n = 103/157) to screen for mitochondrial-related hub genes. Key gene screening was performed by combining three machine learning algorithms—Random Forest, LASSO, and SVM—followed by the construction of a diagnostic neural network model. Additionally, single-cell sequencing data were utilized to analyze the cellular expression patterns of candidate genes in the placenta. To further elucidate the underlying mechanisms, functional validation was conducted both in PE rat model and *in vitro* using HTR-8 cells, supplemented by multi-omics correlation analysis.

**Results:**

Machine learning analysis identified three key genes (GCLM, SNAP23, RHOT2), and the diagnostic model built upon them demonstrated excellent performance (training set AUC = 0.907; validation set AUC = 0.875). Single-cell analysis revealed the expression patterns of these genes within specific cell subtypes, consistent with the transcriptional features of trophoblast cell populations. In the PE rat model, downregulation of GCLM and SNAP23 and upregulation of RHOT2 were significantly correlated with clinical phenotypes such as hypertension and proteinuria, as well as changes in placental inflammatory factor levels (TNF-α, IL-1β, IL-6). Specifically, SNAP23 and GCLM showed negative correlations with inflammatory cytokines but positive correlations with fetal weight, while RHOT2 expression positively correlated with disease severity. *In vitro* experiments confirmed that overexpression of SNAP23 restored mitochondrial membrane potential, reduced reactive oxygen species levels, and suppressed cytokine release in lipopolysaccharide (LPS)-treated HTR-8 cells. Multi-omics analysis further indicated that these genes are involved in immune dysregulation and mitochondrial dysfunction during PE progression.

**Conclusion:**

This study establishes GCLM, SNAP23, and RHOT2 as mechanistically important biomarkers for preeclampsia. Among them, modulation of SNAP23 shows therapeutic potential in alleviating mitochondrial damage and inflammatory responses in PE, providing a new direction for intervention strategies.

## Graphical Abstract

**Figure:**
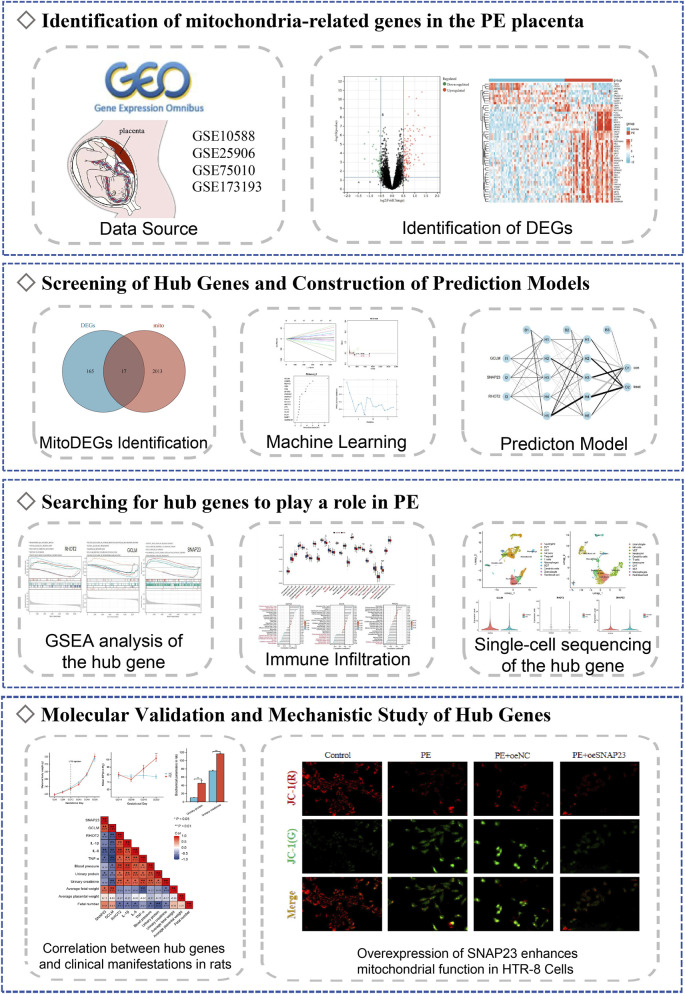

## 1 Introduction

Pre-eclampsia (PE) is a syndrome unique to pregnancy, involving multiple organ systems and impacting around 3%–5% of pregnancies worldwide (Dimitriadis et al., 2023). It is a leading contributor to maternal mortality, causing over 70,000 maternal deaths and more than 500,000 fetal and neonatal deaths globally each year (Wu et al., 2023). Unclear mechanisms, delayed diagnosis, and limited treatment are the main causes of maternal and child mortality (Vaught et al., 2018; Easterling et al., 2019). The etiology of PE is complex, but placental dysfunction is considered to be the core change in the development of PE, and this stage is characterized by oxidative stress, mitochondrial dysfunction, metabolic disorders and apoptosis, but the specific molecular mechanisms are not clear (Dimitriadis et al., 2023; Lyall et al., 2013; Staff et al., 2022). Mitochondria, as major intracellular oxygen consumers, are an important source of elevated reactive oxygen species (ROS) and oxidative stress in PE, thus affecting the progression of PE through various pathways (Hu and Zhang, 2022). For example, abnormalities in mitochondrial function or pathways regulating mitochondrial content in PE placentas result in elevated ROS levels. This may lead to impaired remodeling of the spiral arteries and reduced blood flow to the uteroplacental unit, thus contributing to the development of PE (Long et al., 2023; Padmini et al., 2009). The impaired mitochondrial function plays a key role in PE caused by placental ischemia during pregnancy (Vaka et al., 2018). In addition, blockade of TNF-α, a cytokine strongly associated with the development of PE, significantly improved mitochondrial oxidative stress function in a PE-like rat model (Cunningham et al., 2020). Thus the search for key mitochondrial genes in PE may discover new potential molecular markers.

Dysregulation of the immune microenvironment is another important factor in the development of PE. In normal pregnancy, the maternal immune system is required to develop a tolerance response to exogenous antigens from the fetus, whereas cytokine imbalance, abnormal natural killer cell activity, and impaired regulatory T-cell function are often seen in patients with PE (Deer et al., 2023; Guan et al., 2023; Wei and Yang, 2023). These immunologic changes not only affect the development and function of the placenta, but may also lead to hypertension and other complications. In immune cells, the functional state of mitochondria directly affects cell activity and responsiveness. The generation of ROS is closely related to mitochondrial function, and ROS can promote the activation of immune cells and the release of cytokines, thus affecting the degree of inflammatory response (Zhang et al., 2022). In PE, mitochondrial dysfunction may lead to an abnormal increase in immune cell activity, further exacerbating placental damage and the development of hypertension. The management of PE is currently confined to a limited range of options, with the key strategies being symptom control, complication prevention, and early intervention for the placental-fetal unit (Poon et al., 2019). Thus, there is an urgent need to further elucidate the pathogenesis of PE and advance its diagnostic and therapeutic approaches.

Recent advancements in gene microarray technology and high-throughput genomics have significantly contributed to the efficient identification of differential genes associated with PE (Tesi et al., 2023; Hoheisel, 2006). Additionally, machine learning algorithms offer a promising approach for identifying key biomarkers for PE diagnosis (Handelman et al., 2018). In this study, bioinformatics analysis and machine learning techniques were integrated to study the role of mitochondrial genes in PE, specifically their effects on immune cells and trophoblasts in the placenta. Using placental mRNA sequencing data and single-cell RNA sequencing data from the Gene Expression Omnibus (GEO) database and cell-based experiments, three key candidate driver genes related to mitochondrial metabolism and their possible roles in the pathogenesis of PE were identified, which is helpful in understanding the pathogenesis of PE and improving diagnostic and therapeutic strategies for PE.

## 2 Materials and methods

### 2.1 Data set collection and processing

The PE datasets were obtained from the GEO database (http://www.ncbi.nlm.nih.gov). GSE10588 comprised 26 normal placenta samples and 17 PE placenta samples, while GSE25906 comprised 37 normal placenta samples and 23 PE placenta samples. GSE75010 included 80 normal placenta samples and 77 PE placenta samples.

For our analysis, GSE10588 and GSE25906 were used as the training set, while GSE75010 served as the test set. The training set underwent log-transformation (log (x+1)) normalization, and batch effects were mitigated using the sva package in R. After processing with the ComBat algorithm to remove batch effects, the box plot displays the distribution of the corrected data, highlighting the balance and consistency between different samples. Consequently, we compiled a dataset comprising 63 normal placenta samples and 40 PE placenta samples for further analysis.

### 2.2 Identification of DEGs

The limma package in R was employed to conduct differential gene expression analysis between PE patients and normal controls. Identification of differentially expressed genes (DEGs) using Absolute Log2 Fold Change (FC) > 0.5 and *P* < 0.05 as criteria.

### 2.3 Enrichment analysis

The clusterProfiler package in the R programming environment was used to perform Gene Ontology (GO) enrichment analysis and Kyoto Encyclopedia of Genes and Genomes (KEGG) pathway enrichment analysis. The GO enrichment maps from the annotation analysis were visualized using the “ggplot2” and “GOplot” packages, and a corrected *P* < 0.05 was selected to identify significantly enriched pathways.

### 2.4 Screening of hub genes

The list of mitochondria-related genes was sourced from the MitoCarta 3.0 database (https://www.broadinstitute.org/mitocarta) and MSigDB database (http://www.gsea-msigdb.org/gsea). A total of 2030 mitochondria-related genes were collected, and the specific list of genes is detailed in Additional Table 1.

**TABLE 1:** The primer set for associated mRNA.

Gene name	Forward primer (5′–3′)	Reverse prime (5′–3′)	Fragment length (bp)
RHOT2	CTA​CTC​AGA​AGC​CGA​GCA​GAC​G	TTCTCAATGGCCTCCTCA	105
GCLM	GTG​ATG​CCA​CCA​GAT​TTG​ACT​G	CAC​TCG​TGC​GCT​TGA​ATG​TC	143
SNAP23	AAT​CCT​GGG​TTT​AGC​CAT​TGA​G	TGC​GGT​TTA​GTT​GTT​CCT​TTT​G	86
TBP	TGC​ACA​GGA​GCC​AAG​AGT​GAA	CAC​ATC​ACA​GCT​CCC​CAC​CA	112

Subsequently, the DEGs identified previously were intersected with the mitochondria-related genes to obtain a subset termed MitoDEGs. These MitoDEGs represent genes that are both differentially expressed and involved in mitochondrial function, potentially playing critical roles in the development of PE. The R package pheatmap was utilized to draw heatmaps visualizing MitoDEG expression patterns. Based on the differentially expressed gene matrix, multi-dimensional feature screening was performed using Random Forest (RF), LASSO regression (Least Absolute Shrinkage and Selection Operator), and Support Vector Machine (SVM). Finally, the GSE75010 dataset was selected as an external validation set to further verify the genes identified through machine learning. Genes exhibiting consistent differential expression trends across analyses were chosen for subsequent investigation. These persistently expressed genes were defined as hub genes.

### 2.5 Single gene GSEA analysis

To investigate the relationship between hub genes and biological processes, we divided the PE samples into a high-expression group and a low-expression group based on the median expression level of the hub genes. Following this classification, we conducted GSEA on the subgroups, using a significance level of FDR <0.05.

### 2.6 ANN construction

A biomarker model based on an Artificial Neural Network (ANN) was developed in the R software environment using the neuralnet and neuralnettools packages. The model architecture comprised two hidden layers, each containing five nodes. Leveraging “gene score” information, a classification model was constructed to distinguish between the normal and disease groups. The predictive performance of the ANN was evaluated through calibration curves, decision curve analysis (DCA), and Receiver Operating Characteristic (ROC) curves of the diagnostic model.

### 2.7 Immune infiltration analysis

ssGSEA, enabled by the Gene Set Variation Analysis (GSVA) package in R, is a technique developed to quantify gene set enrichment within individual samples. Specifically, ssGSEA measures the extent of infiltration of 28 different immune cell types and detects changes in these gene sets between the PE and control groups. Spearman correlation analysis was employed to explore potential associations between hub genes and infiltrating immune cells, aiming to determine their correlation.

### 2.8 Analysis of single-cell RNA-seq data

The PE placental single-cell sequencing dataset (GSE173193) was obtained from the GEO database. The scRNA-seq dataset underwent analysis using “Seurat” and “SingleR”. Filter cells according to criteria including nFeature RNA (≤200 or ≥2000) and mitochondrial percentage (>5%) to remove low quality cells. Subsequently, doublets were identified and removed using scrulet to ensure result reliability. Downscaling and clustering analyses were performed, and 3000 highly variable genes were selected. Utilizing elbow diagrams, principal components (PCs) with inflection points and smooth curves were chosen, and 20 dimensions were selected for analysis to illustrate the downscaling effect of UMAP and tSNE. The unique genes of each dimension were pooled for annotation using the cellmaker database. Finally, violin plots and co-localization mapping were employed to illustrate the expression of hub genes across different cellular subpopulations.

### 2.9 Animal experimental validation of LPS-Induced model construction

The preeclampsia-like rat model was established via intraperitoneal (IP) injection of LPS. Specifically, 20 μg/kg LPS was administered daily through IP injection from gestational day (GD) 13 to GD 18 for model construction (Jahan et al., 2023). The control group received equivalent doses of normal saline through IP injection. Non-invasive blood pressure measurements were performed on GD 14, 16, 18, and 20. On GD 19 evening, rats were placed in metabolic cages for urine collection to assess renal function through urinary protein and creatinine measurements. After the rats were terminated, the uterus was quickly excised to record fetal count, fetal weight, and placental weight. Subsequently, placentas were swiftly dissected, rinsed with pre-chilled physiological saline (4 °C), gently blotted on filter paper, and weighed—with all steps carried out on ice to prevent RNA degradation. All procedures were performed on ice to minimize RNA degradation. After weighing, each placenta was segmented, transferred into pre-chilled RNase-free cryotubes, and immediately snap-frozen in liquid nitrogen (Jahan et al., 2023). Placental tissues from rats were utilized to analyze the expression patterns of key genes and the concentrations of inflammatory cytokines. The study protocol was strictly reviewed and approved by the Laboratory Animal Ethics Committee of Henan University of Chinese Medicine, with the ethical approval number DWLL201903027.

### 2.10 Cell culture and treatment

HTR-8/SVneo cells were generously provided by Dr. Jianhua Sun. The cells were cultured in RPMI 1640 medium (Servicebio, China, G4538) containing 10% fetal bovine serum (Corning, China, 26219003) and 100 U/mL penicillin-streptomycin (Servicebio, G4003) at 37 °C in a 5% CO2 incubator. To mimic PE disease, different concentrations of LPS were used for intervention (50, 100, 200, 400, 800 ng/mL; Servicebio, GC205009). Cellular activity was then assayed using CCK-8, and the lowest concentration with a significant effect was selected to mimic PE disease.

### 2.11 RNA extraction and quantitative real-time PCR (qRT-PCR)

Total RNA was extracted using Trizol reagent (Servicebio, G3013). RT-qPCR was used to measure expression levels of RHOT2, GCLM, and SNAP23. Relative changes in mRNA levels were determined using the 2^−ΔΔCt^ method. RT-qPCR conditions were: 95 °C for 30 s, 40 cycles of 95 °C for 15 s, 60 °C for 30 s, and a final step from 60 °C for 60 s to 95 °C for 15 s. TBP was used as a housekeeping gene to normalize target gene expression. The primers used are shown in Table 1.

### 2.12 ROS levels and MMP detection

HTR-8 cells were inoculated into 24-well plates at a density of 1 × 10^5^ cells per well. Twenty-four hours after inoculation, the intervention was performed according to the predetermined grouping. After an additional 24 h, changes in mitochondrial membrane potential (MMP) and ROS levels were detected in different groups.

MMP was assessed using JC-1 staining (Servicebio, G1515). Intracellular ROS levels were detected using the DCFH-DA fluorescent probe (Servicebio, G1706). Following incubation, cells were examined under a fluorescence microscope. Mitochondrial MMP and ROS levels were quantified using ImageJ software.

### 2.13 Enzyme-linked immunosorbent assay (ELISA)

The concentrations of inflammatory cytokines (including TNF-α, IL-8, and IL-6) in animal placental tissues and cell culture supernatants were measured using a commercially available ELISA kit (Mlbio, China).

### 2.14 RNA interference

Control plasmids and plasmids mediating SNAP23 overexpression were obtained from Unibio (Unibio, China). In the LPS induction group, cells were transfected using Lipofectamine 2000 transfection reagent (Invitrogen, Carlsbad, CN2478853) when they reached approximately 70% confluence, 24 h post-induction. The transfected cells were then used for subsequent experiments after an additional 24 h.

### 2.15 Statistical analysis

All analyses were conducted using R (version 4.2.2). To compare two groups, either the parametric Student’s t-test or the non-parametric Mann-Whitney test was used. Analyses involving more than two groups utilized one-way ANOVA, with subsequent Bonferroni post-hoc testing for significance. Statistical significance was defined as *P* < 0.05.

## 3 Result

### 3.1 Identification of DEGs

**FIGURE 1:**
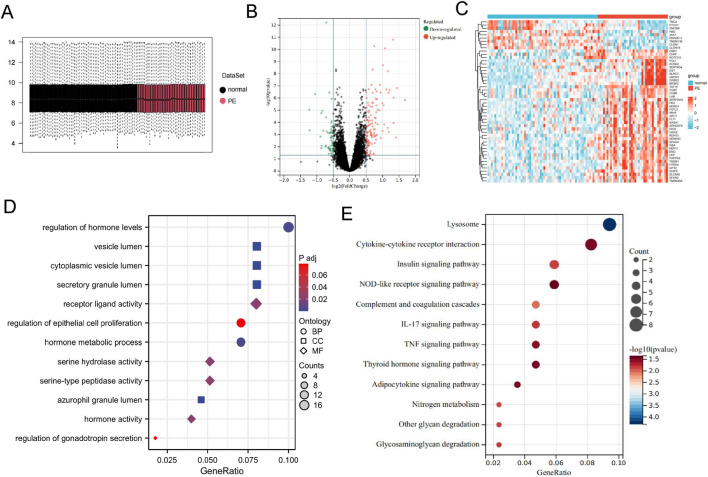
Identification of the DEGs in PE. **(A)** Box plot after removing batch effects using the ComBat algorithm. **(B)** Volcano plot of DEGs. **(C)** Heatmap of DEGs. **(D)** GO enrichment analysis. **(E)** KEGG enrichment analysis.

Microarray datasets GSE10588 and GSE25906 were retrieved from the GEO database, comprising 63 samples from the normal group and 40 samples from the PE disease group. The box plot demonstrates the successful removal of batch effects by the ComBat algorithm (Figure 1A). This consistency indicated successful mitigation of any potential batch effects. Through differential analysis, a total of 182 DEGs were identified. Among these, 125 genes exhibited significant upregulation, while 57 genes showed significant downregulation (Figures 1B,C).

### 3.2 Functional enrichment analysis

KEGG analysis revealed that differentially expressed genes were enriched in pathways associated with lysosomes, the complement system, energy metabolism, and cytokines, with multiple pathways demonstrating significant correlations with mitochondrial function (Figure 1D). In GO enrichment analysis, Cellular Component (CC) terms were predominantly focused on vesicle lumen, cytoplasmic vesicle lumen, and secretory granule lumen. Biological Process (BP) terms mainly centered around regulation of hormone levels, regulation of epithelial cell proliferation, hormone metabolic processes, and regulation of gonadotropin secretion. Molecular Function (MF) terms primarily highlighted receptor ligand activity, serine hydrolase activity, and serine-type peptidase activity (Figure 1E).

### 3.3 Identification of mitochondria-related genes in PE

**FIGURE 2:**
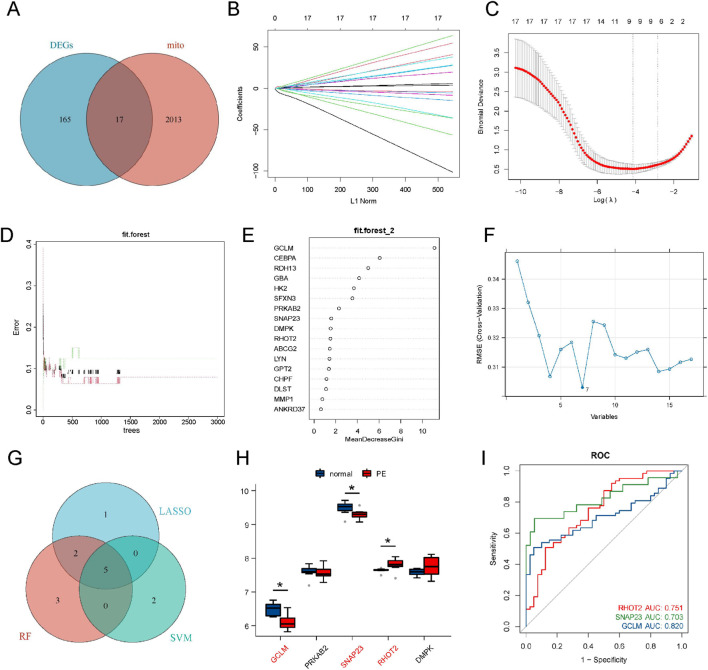
Identification of hub gene. **(A)** Venn diagram of DEGs and mitochondria-related genes; **(B,C)** Screening of hub genes using the LASSO algorithm; **(D,E)** Screening of hub genes using the RF algorithm; **(F)** SVM decision boundary plot; **(G)** Intersection Venn diagram of RF, LASSO, and SVM; **(H)** Validation with the external dataset GSE75010; **(I)** ROC curves of hub genes in the experimental cohort.

After intersecting the 182 DEGs with 2030 mitochondrial genes from the MitoCarta 3.0 and MSigDB databases, 17 overlapping genes, referred to as mitoDEGs, were identified (Figure 2A). The differentially expressed genes related to mitochondria in PE were further screened by LASSO regression, SVM and RF methods. In the LASSO regression analysis, eight candidate genes were screened out by 10-fold cross-validation to select the optimal regularization parameter (Figures 2B,C). When the ntree parameter of the Random Forest was set to 1000, the error rate tended to be stable. At this time, the top 10 genes were selected according to the MeanDecreaseGini ranking (Figures 2D,E). For the SVM, the Recursive Feature Elimination (SVM-RFE) method was adopted for feature selection. By plotting the decision boundary diagrams of different feature subsets, it was observed that when the number of features was reduced to 7, the decision boundary reached the optimal separation state, and the genes screened at this time were selected as candidates (Figure 2F). By taking the intersection through the Venn diagram, five overlapping genes (GCLM, CEBPA, RDH13, GBA, HK2, SNAP23, RHOT2) were finally determined (Figure 2G).

**FIGURE 3:**
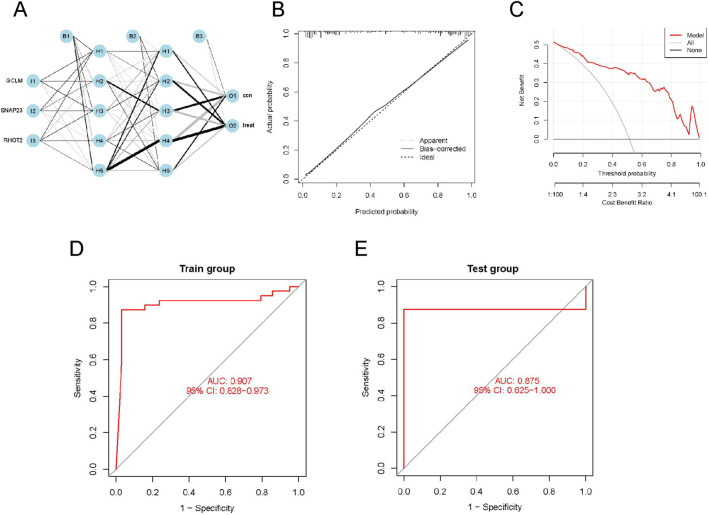
ANN model construction based on hub genes. **(A)** ANN model; **(B)** ANN model calibration curve; **(C)** ANN model diagnostic curve; **(D)** ROC curves of the Artificial Neural Network on the Train group; **(E)** ROC curves of the Artificial Neural Network on the Test group.

Subsequently, GSE75010 was utilized as an external dataset to validate the accuracy of the five genes identified. Among these, three genes (SNAP23, GCLM, RHOT2) exhibited consistent performance with the training set, thereby establishing them as reliable diagnostic biomarkers (Figures 3H,I).

### 3.4 Predictive modeling of hub genes with ANN

Utilizing three previously identified key genes, a gene weight-based diagnostic model was constructed using an ANN (Figure 3A). The model’s accuracy and clinical utility were jointly validated through calibration curves, decision curve analysis (DCA), and ROC curve analysis. The calibration curve demonstrated high concordance between predicted and observed risks (slope approaching 1), indicating excellent calibration of the model’s predicted probabilities (Figure 3B). DCA revealed a significantly higher net benefit across a wide threshold probability range compared to “treat-all” and “treat-none” strategies, confirming the model’s value in clinical decision-making (Figure 3C). ROC curve analysis further showed outstanding discriminatory power in both the training set (AUC = 0.907) and validation set (AUC = 0.875) (Figures 3D,E). Together, these results comprehensively demonstrate the model’s high accuracy, reliability, and clinical applicability for the diagnosis of PE.

### 3.5 GSEA analysis of the hub gene

**FIGURE 4:**
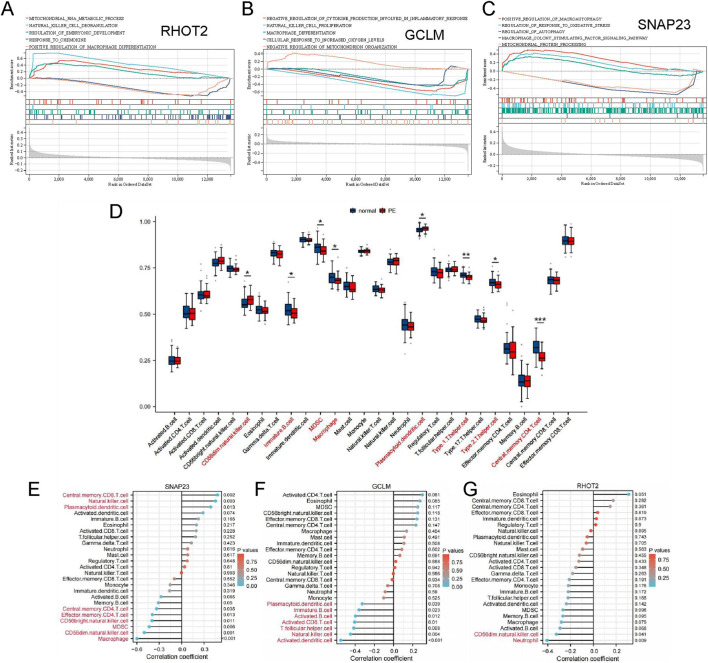
GSEA and immune infiltration analysis. **(A–C)** GSEA analysis of RHOT2, GCLM, and SNAP23; **(D)** Comparison of immune cell infiltration between PE and controls; **(E)** The association between SNAP23 and different immune cell infiltration in PE. **(F)** The association between GCLM and different immune cell infiltration in PE.; **(G)** The association between RHOT2 and different immune cell infiltration in PE. *: *p* < 0.05; **: *p* < 0.01; ***: *p* < 0.001.

Based on GSEA analysis, we identified potentially relevant pathways associated with the three central genes, some of which are linked to PE. RHOT2 demonstrated significant associations with pathways such as positive regulation of macrophage differentiation and response to chemokine (Figure 4A). GCLM showed significant associations with pathways such as negative regulation of cytokine production involved in inflammatory response and Macrophage differentiation (Figure 4B). SNAP23 exhibited significant associations with pathways including positive regulation of macroautophagy and regulation of response to oxidative stress (Figure 4C). These results underscore the pivotal role of these three genes in the onset of preterm PE, providing valuable insights into understanding the underlying mechanisms of the condition.

### 3.6 Immune infiltration analysis

The CIBERSORT algorithm is commonly used to analyse and compare immune cell infiltration in disease groups and healthy controls (Zhao et al., 2023). The analysis uncovered notable disparities in immune cell infiltration between PE patients and normal controls. Notably, CD56dim Natural Killer (NK) cells and plasmacytoid dendritic cells were markedly elevated in PE patients. Conversely, in the normal group, immature B cells, myeloid-derived suppressor cells (MDSCs), central memory CD4 T cells, macrophages, Type 1 T helper cells, and Type 2 T helper cells were significantly elevated (Figure 4D).

### 3.7 Analyzing the correlation between hub genes and infiltrating immune cells

Immune infiltration analysis of placental tissues from patients with PE revealed intriguing interactions between hub genes and distinct immune cell types. SNAP23 exhibited positive associations with NK cells, plasmacytoid dendritic cells, and central memory CD8 T cells. Conversely, it showed positive associations with activated B cells, CD56bright NK cells, CD56dim NK cells, myeloid-derived suppressor cells (MDSCs), macrophages, effector memory CD4 T cells, and central memory CD4 T cells, while negatively correlating with GCLM and T follicular helper cells (Figure 4E). GCLM displayed negative correlations with activated B cells, activated CD8 T cells, activated dendritic cells, immature B cells, NK cells, plasmacytoid dendritic cells, and T follicular helper cells (Figure 4F). RHOT2 showed a negative correlation with CD56dim NK cells and neutrophils (Figure 4G).

### 3.8 Single-cell analysis of the hub gene

**FIGURE 5:**
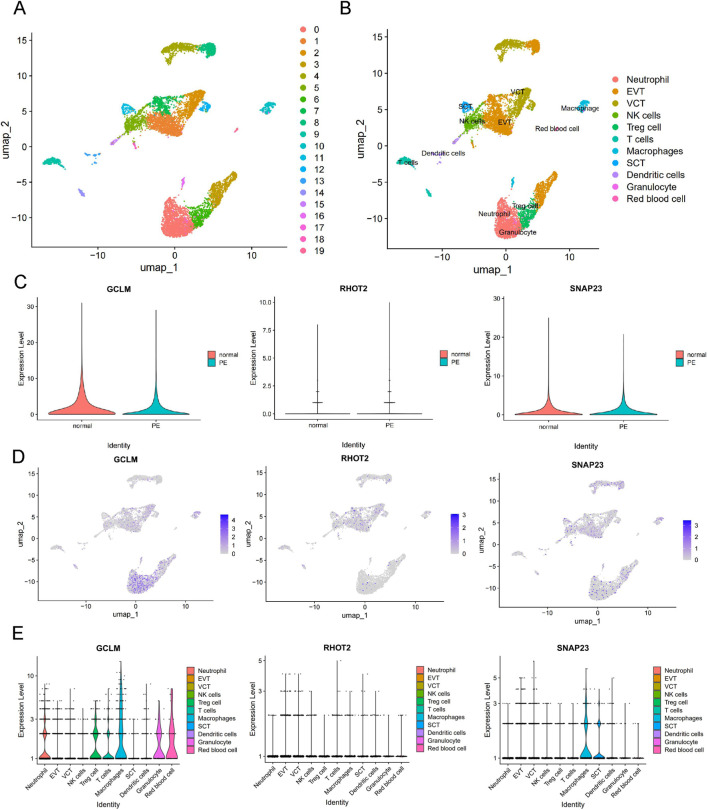
Distribution and expression of hub genes based on single-cell RNA sequencing data. **(A)** UMAP display of single-cell grouping in PE patients; **(B)** PE patients categorized into 11 cell subsets; **(C)** Violin plots depicting hub gene expression in normal and PE patients; **(D)** FeaturePlot illustrating the distribution and expression of hub genes within the PE cell population; **(E)** Violin plots showing hub gene expression in PE cells. Villous cytotrophoblast cell (VCT), Syncytiotrophoblast cell (SCT), Extravillous trophoblast cells (EVT).

Single-cell analysis of placental data from GSE173193 revealed a total of 11 cell populations (Figures 5A,B). The single-cell annotation map for the control group is shown in Appendix Figure 1. Consistent with previous analyses in this study, the PE group exhibited a trend towards decreased GCLM and SNAP23 expression and increased RHOT2 expression (Figure 5C). Further analysis of central gene expression in various cell types using FeaturePlot and violin plots (Figures 5D,E) revealed that GCLM was broadly expressed across immune cells, while SNAP23 was primarily found in macrophages and syncytiotrophoblast cells (SCT), with lower levels in other cell types. In contrast, RHOT2 showed low expression across various cell types.

**FIGURE 6:**
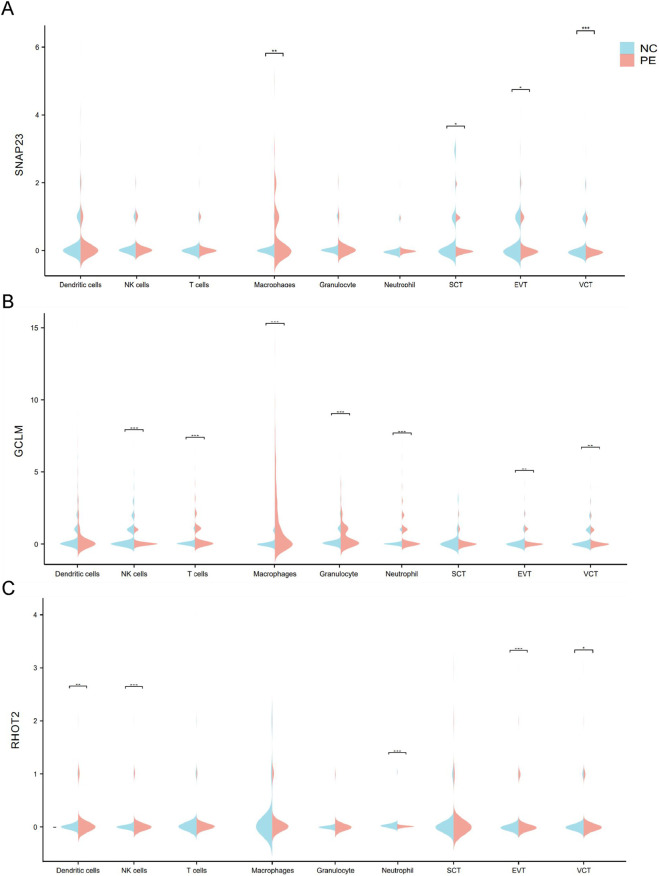
Analysis of hub gene expression across different cell clusters in both PE and normal patients. **(A)** Violin plot depicting SNAP23 gene expression in each cell cluster; **(B)** Violin plot illustrating GCLM gene expression in each cell cluster; **(C)** Violin plot displaying RHOT2 gene expression in each cell cluster. *: *p* < 0.05; **: *p* < 0.01; ***: *p* < 0.001.

Violins were plotted by quantitatively analyzing SNAP23, GCLM, and RHOT2 expression in PE and normal groups. SNAP23 expression was increased in macrophages but decreased in all three trophoblast types (Figures 6A–C). GCLM expression increased in NK cells, macrophages, granulocytes, neutrophils, and other immune cells, but decreased in SCT and extravillous trophoblast cells (EVT). RHOT2 expression showed a clear downward trend in dendritic cells and NK cells, while it increased in EVT and villous cytotrophoblast cells (VCT). In conclusion, an interesting phenomenon was observed: the expression of SNAP23, GCLM, and RHOT2 in various immune and trophoblast cells in the placenta mostly exhibited opposite trends. This may be related to the inconsistent expression of immune cell and trophoblast mitochondrial function after the onset of PE.

### 3.9 Molecular validation of hub genes

**FIGURE 7:**
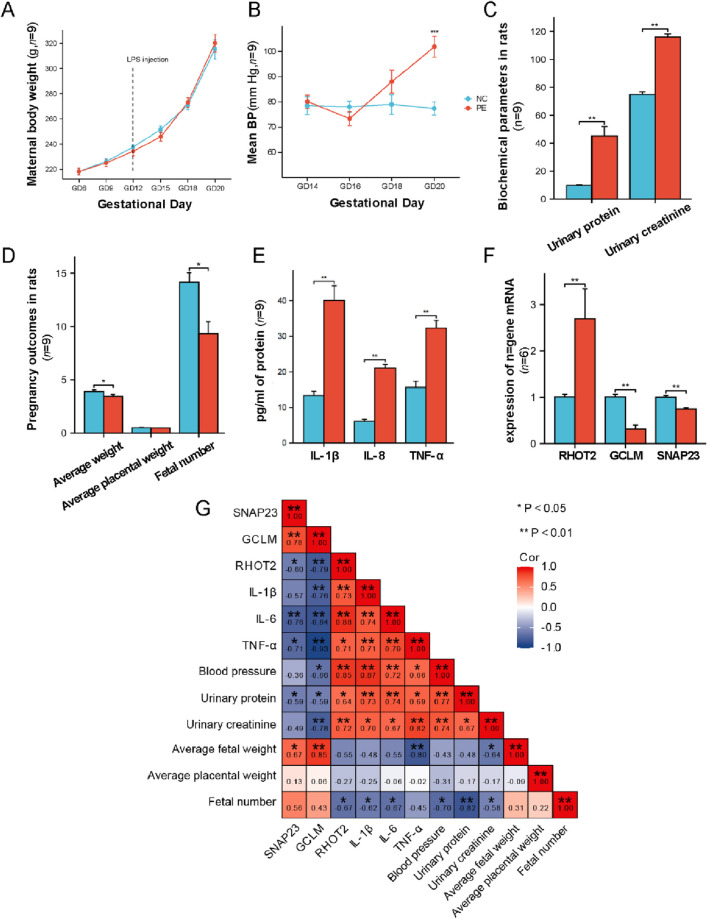
Validation of animal experiments constructed by LPS. **(A)** Body weight changes in the two rat groups from GD6 to GD20; **(B)** Blood pressure changes in the two rat groups from GD14 to GD20; **(C)** Biochemical parameters of rats; **(D)** Pregnancy outcome parameters of rats; **(E)** Expression levels of inflammatory factors in rat placental; **(F)** Expression levels of hub genes in rat placenta; **(G)** Correlation analysis. *: *p* < 0.05; **: *p* < 0.01; ***: *p* < 0.001.

During rat pregnancy, body weight changes were continuously monitored, with measurements taken on GD 6, 9, 12, 15, 18, and 20. No statistically significant differences in maternal weight were observed between groups (Figure 7A). Continuous blood pressure monitoring revealed a significant increase in the PE group compared to NC group on GD20 (Figure 7B). Urinary protein quantification showed significantly elevated protein levels in LPS-induced PE rats on GD20, and urinary creatinine measurements confirmed a marked increase in proteinuria in the PE group at the same time point (Figure 7C). The PE group displayed a significant decrease in fetal number and fetal weight, although average placental weight remained unchanged (Figure 7D). In terms of inflammatory factors, placental levels of TNF-α, IL-1β, and IL-6 were significantly elevated in the PE group compared to the control group (Figure 7E). RT-qPCR results aligned with sequencing data: CCL2 and CX3CL1 expression levels were significantly upregulated in PE rats, while GCLM expression was markedly downregulated compared to the normal group (Figure 7F).

Spearman correlation analysis of mitochondrial-related key gene expression, placental inflammatory factors, pregnancy outcomes, blood pressure, urinary protein, and creatinine levels revealed that SNAP23 expression was significantly negatively correlated with IL-6, TNF-α, and proteinuria, while showing a significant positive correlation with average fetal weight. GCLM expression demonstrated significant negative correlations with IL-1β, IL-6, TNF-α, blood pressure, proteinuria, and creatinine levels, but a significant positive correlation with average fetal weight. RHOT2 expression exhibited significant positive correlations with IL-1β, IL-6, TNF-α, blood pressure, proteinuria, and creatinine levels, as well as a positive correlation with fetal number (Figure 7G).

### 3.10 Mechanistic study of hub genes

**FIGURE 8:**
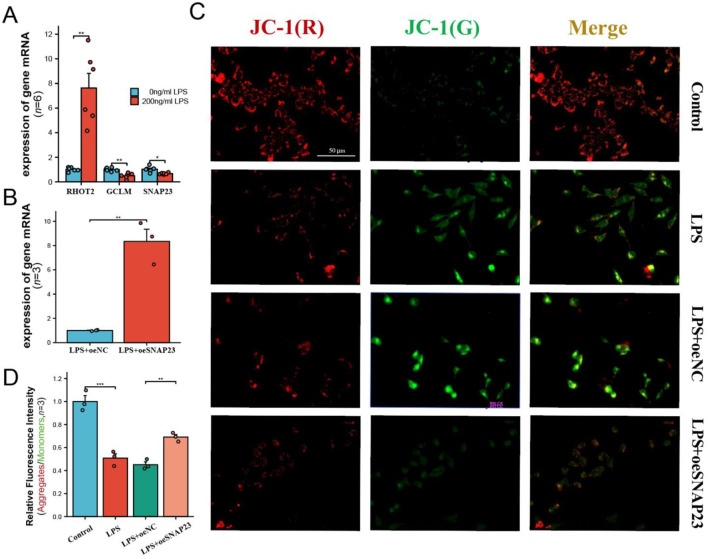
MMP levels in HTR-8 cells. **(A)** Expression of GCLM, RHOT2, and SNAP23 in HTR-8 cells treated with 0 ng/ml LPS and 200 ng/ml LPS concentrations. **(B)** SNAP23 RNA interference assay. **(C)** MMP levels in HTR-8 cells were detected using JC-1 fluorescent probe. **(D)** Relative aggregates/monomers fluorescence intensity ratio in HTR-8 cells. *: *p* < 0.05; **: *p* < 0.01; ***: *p* < 0.001.

**FIGURE 9:**
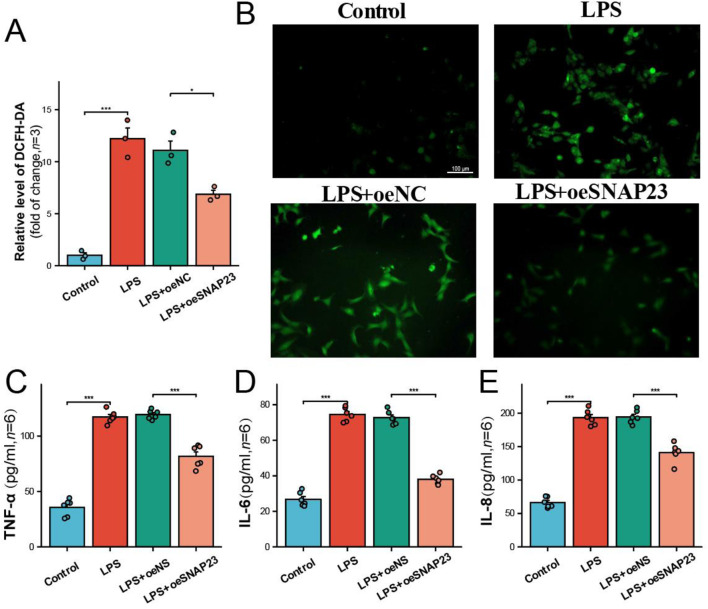
Levels of ROS and inflammatory factors in HTR-8. **(A)** Statistical results of relative ROS fluorescence in HTR-8 cells. **(B)** ROS levels were detected in HTR-8 cells using the DCFH-DA fluorescent probe. **(C–E)** Comparison of inflammatory factor release between different groups. *: *p* < 0.05; **: *p* < 0.01; ***: *p* < 0.001.

Based on the CCK-8 experiment results, a concentration of 200 ng/mL LPS was selected to simulate PE. The expression of three pivotal mitoDEGs in LPS-treated HTR-8 cells was confirmed using RT-PCR. Compared with controls, GCLM and SNAP23 were significantly reduced, whereas RHOT2 expression was significantly elevated. This is consistent with previous placental mRNA sequencing results (Figure 8A). To further explore the function of hub genes, and building on previous single-cell analyses, we selected SNAP23 for RNA interference experiments (Figure 8B). JC-1 staining revealed reduced mitochondrial membrane potential in the PE group, which was improved by SNAP23 overexpression (Figures 8C,D). DCFH-DA staining showed increased ROS in the PE group, also reversed by SNAP23 overexpression (Figures 9A,B). ELISA results indicated that LPS increased TNF-α, IL-6, and IL-8 levels, which were attenuated after SNAP23 overexpression (Figures 9C–E).

## 4 Discussion

PE is a significant contributor to adverse perinatal outcomes for both mother and baby. Currently, the only effective intervention is timely termination of pregnancy, and therefore there is an urgent need to enhance our understanding of PE pathogenesis and identify innovative therapeutic targets. In this study, we used multiple sequencing data to identify three key mitochondria-related regulatory genes (SNAP23, GCLM, RHOT2) in PE, and in particular, the changes of RHOT2 in PE were not reported. At the same time, they were used to establish high-performance diagnostic models, and their molecular mechanisms were clarified through multiple biometric analysis.

We searched for differential genes and conducted enrichment analysis by integrating PE-related clinical data from the GEO database, finding that the enriched pathways were primarily associated with energy metabolism and inflammatory factors. Given that mitochondrial abnormalities play a key role in PE by impacting both energy metabolism and inflammation, we focused our analysis on mitochondria-related genes in PE. While studies have identified mitochondrial-related differentially expressed genes in preeclampsia, their functional roles are not yet fully elucidated. Further research is essential to better understand the disease’s pathology (Li et al., 2025; Bartho et al., 2022; Huang et al., 2024). PE-specific differential genes were identified by integrating PE-related data from the GEO database and crossing them with a collection of mitochondria-related genes. This approach yielded a total of 17 mitochondria-related DEGs, which were subsequently selected for further analysis. Three biomarkers were successfully identified through the application of multiple machine learning techniques and external datasets. GCLM (Glutamate cysteine ligase regulatory subunit), serves as a pivotal regulatory element of oxidative stress-associated glutamate-cysteine ligase. It plays a critical role in regulating oxidative stress, preventing its escalation, and safeguarding mitochondrial functional integrity (Plačková et al., 2016; Ran et al., 2022; Zhang et al., 2011). Recent findings indicate that serum glutamate-cysteine ligase (GCL) levels are markedly reduced in PE patients compared to healthy pregnant women (Mazloomi et al., 2021). Furthermore, separate experimental analyses have identified GCLM as a novel diagnostic biomarker for PE (Tan et al., 2026). Levels of its related product, glutathione, are markedly altered in PE, suggesting disrupted glutathione metabolism in placental tissues as a potential pathogenic mechanism in PE development (Jin et al., 2017). Additionally, overexpression of GCLM has been shown to mitigate TNF-induced mitochondrial damage and reduce levels of ROS, aligning with current research indicating that low expression of GCLM correlates with impaired mitochondrial function and oxidative stress in PE (Zhang et al., 2011).

RHOT2 (Mitochondrial Rho GTPase 2), a mitochondrial transport protein belonging to the Rho family of GTPases, resides in the outer mitochondrial membrane and plays a critical function in the articulators complex, which is essential for tethering mitochondria to motor proteins. This protein is essential for maintaining mitochondrial homeostasis and controlling apoptosis (Safiulina et al., 2018; Schwarz, 2013; König et al., 2021; Fransson et al., 2003). Current understanding suggests that RHOT2 binds to Ca2+, thereby inhibiting mitochondrial mobility, whereas normal mobility is essential for preserving mitochondrial function and morphology (Wang and Schwarz, 2009; Zhang et al., 2021). Currently, there are no reported changes in RHOT2 in PE. Our study revealed increased RHOT2 levels in PE trophoblasts, which may indicate reduced mitochondrial activity. This impairment in motility is considered a contributing factor to the mitochondrial dysfunction and apoptosis seen in preeclampsia (LeFort et al., 2024; El-Hattab and Scaglia, 2016).

SNAP23 (Encoding Synaptosome-associated Protein 23), belongs to the SNARE (Soluble N-Ethylmaleimide Sensitive Factor Attachment Protein Receptor) family pivotal for intracellular transport and secretion. It plays a pivotal role in macrophage phagocytosis and regulates cytokine secretion, adapting to immune activation changes through modulation of membrane trafficking and cytokine release onset (Veale et al., 2011; Hatsuzawa and Sakurai, 2020; Pagan et al., 2003). In PE, there is a notable shift in macrophage polarization, characterized by an increased proportion of M1-type macrophages (Leavey et al., 2019). This polarization process appears to be influenced by SNARE proteins. SNAP23, downstream of glucose transporter 4 (GLUT4), facilitates vesicle and plasma membrane fusion, accelerating glucose transport (Kioumourtzoglou et al., 2017; Zhao et al., 2021; Senthivinayagam et al., 2013). Reduced SNAP23 expression in PE placenta suggests impaired glucose transporter function, exacerbating tissue energy metabolism stress, potentially leading to programmed apoptosis. It has been demonstrated that knockdown of SNAP23 in rats results in severe generalized lipodystrophy and inflammation of adipose tissue. This adipose tissue inflammation leads to the release of adipocytokines, which in turn leads to the development of PE, as reflected in the KEGG enrichment analysis we performed previously (Hendler et al., 2005; Dalamaga et al., 2011; Kelly et al., 2017).

Subsequently, we further investigated the potential mechanisms of key genes through single-gene GSEA, immune infiltration analysis, and single-cell transcriptomic analysis. It was found that three mitochondrial-related hub genes—GCLM, RHOT2, and SNAP23—were significantly associated with PE-characteristic immune cell phenotypes, including aberrant macrophage polarization, dysfunctional NK cell activity, and neutrophil activation. Specifically, RHOT2 may drive PE progression by positively regulating macrophage differentiation and chemokine response pathways; GCLM likely exerts protective effects by negatively modulating inflammation-related cytokine production pathways; and SNAP23 may participate in disease pathogenesis via regulation of macroautophagy activation and oxidative stress response pathways. However, the specific molecular mechanisms and spatiotemporal specificity of these regulatory networks require further validation using conditional gene knockout models and single-cell spatiotemporal omics technologies. Single-cell resolution analysis of key genes in PE revealed that the expression patterns of SNAP23, GCLM, and RHOT2 in placental trophoblast cells aligned with overall trends, suggesting their regulation is primarily influenced by trophoblast-specific expression. Intriguingly, their expression trends varied across immune cell subtypes, potentially indicating altered mitochondrial functions in immune cells during PE progression or other regulatory influences requiring further investigation. These findings strongly emphasize the potential interplay between these hub genes, placental trophoblast cells, and immune cells, underscoring their pivotal roles in PE pathogenesis.

In summary, our study used machine learning techniques to identify three key genes (GCLM, SNAP23, RHOT2) that regulate the critical processes of macrophage polarization and differentiation, macrophage chemotaxis, natural killer cell proliferation, and activation of the inflammatory response. These processes are intricately linked to mitochondrial function. These genes are thought to play key roles in the development and progression of PE by influencing mitochondrial oxidative stress, regulating macrophage function, and triggering inflammatory responses. Targeting these genes may slow the progression of PE by attenuating these underlying pathological processes. Regrettably, we only verified the effects of these three genes on trophoblasts and did not investigate the crosstalk between different cell types in placental tissue.

Experimental validation in a PE rat model demonstrated correlations between the expression levels of GCLM, SNAP23, and RHOT2 and the severity of placental inflammation (IL-1β, IL-6, TNF-α), adverse pregnancy outcomes (average fetal weight, fetal number), and PE-like symptoms (GD20 blood pressure, urinary protein, and creatinine), suggesting their synergistic involvement in PE pathogenesis. Based on single-cell transcriptomic findings, further experiments in the HTR-8 trophoblast cell line revealed that modulating SNAP23 expression improved mitochondrial function in LPS-treated trophoblasts, reduced oxidative stress, and attenuated inflammatory cytokine release, highlighting its potential as a therapeutic target for PE. However, this study only verified the effects of these genes on trophoblast cells, and the findings cannot be directly extrapolated to all placental cell types. Moreover, interactions between different placental cell types were not explored. These complex interrelationships and their underlying mechanisms still need to be further elucidated through subsequent experiments.

Additionally, this study proposes a prediction model based on placental sequencing data, though challenges remain in translating placenta-derived biomarkers into effective early pregnancy screening tools. Currently, there are various clinical prediction models for PE, utilizing markers such as vascular endothelial growth factor (VEGF), soluble endoglin (sEng), and soluble tyrosine kinase 1 (sFlt-1) (Melinte-Popescu et al., 2023; Loftness et al., 2023; Ansbacher‐Feldman et al., 2022). A recent study used cell-free RNA (cfRNA) profiling to noninvasively predict PE (Moufarrej et al., 2022). The non-invasive nature and broad applicability of cfRNA in prenatal screening underscore its considerable clinical promise. Analysis of cfRNA expression profiles may provide a platform for the earlier and more specific of conditions including preeclampsia, potentially guiding preemptive clinical management. Technical standardization and rigorous clinical validation, nevertheless, are prerequisites for its routine implementation. Mitochondria-associated genes GCLM, RHOT2, and SNAP23 are expressed in various immune cells within the bloodstream. However, whether their expression levels in the blood of PE patients align with the trends observed in placental expression remains to be determined. Further clinical research is needed to validate these findings.

Moreover, increasing the cohort size could enhance the accuracy of assessing and predicting PE. Further validation of the identified biomarkers in animal models is crucial to provide a solid foundation for clinically targeted therapies. Despite recent advances in understanding the interactions between inflammation and mitochondria in PE, more research is needed. Specifically, examining the correlation between macrophage polarization, natural killer cell proliferation, and inflammation in PE requires further attention and will help us to more fully understand the disease process in PE.

## 5 Conclusion

We investigated mitochondrial metabolic function in PE patients and identified three mitochondrial hub genes (GCLM, SNAP23, and RHOT2) through transcriptomic and single-cell sequencing data analyses. An ANN model constructed based on these genes demonstrated high accuracy and reliability. The expression levels of GCLM, SNAP23, and RHOT2 were significantly correlated with placental inflammation, adverse pregnancy outcomes, and the severity of PE-like symptoms in rats. Furthermore, modulating SNAP23 expression has been demonstrated to reverse abnormal mitochondrial membrane potential, elevated ROS levels, and the release of inflammatory cytokines.

## Data Availability

The original contributions presented in the study are publicly available. These data can be found in the NCBI Gene Expression Omnibus (GEO) repository under the accession numbers GSE10588, GSE25906, GSE75010, and GSE173193.
